# A machine learning model in predicting hemodynamically significant coronary artery disease: A prospective cohort study

**DOI:** 10.1016/j.cvdhj.2022.02.002

**Published:** 2022-03-07

**Authors:** Yan Liu, Haoxing Ren, Hanna Fanous, Xuming Dai, Hope M. Wolf, Tyrone C. Wade, Cassandra J. Ramm, George A. Stouffer

**Affiliations:** ∗ Dell Medical School, The University of Texas at Austin, Austin, Texas; † Division of Cardiology, Department of Medicine, School of Medicine, University of North Carolina at Chapel Hill, Chapel Hill, North Carolina; ‡ Engine Medical Software, Austin, Texas; § New York Presbyterian Medical Group–Queens, New York, New York; ‖ Department of Human and Molecular Genetics, Virginia Commonwealth University School of Medicine, Richmond, Virginia

**Keywords:** Coronary artery disease, Machine learning, Artificial intelligence, Health care efficiency

## Abstract

**Background:**

Coronary artery disease (CAD) costs healthcare billions of dollars annually and is the leading cause of death despite available noninvasive diagnostic tools.

**Objective:**

This study aims to examine the usefulness of machine learning in predicting hemodynamically significant CAD using routine demographics, clinical factors, and laboratory data.

**Methods:**

Consecutive patients undergoing cardiac catheterization between March 17, 2015, and July 15, 2016, at UNC Chapel Hill were screened for comorbidities and CAD risk factors. In this pilot, single-center, prospective cohort study, patients were screened and selected for moderate CAD risk (n = 185). Invasive coronary angiography and CAD prediction with machine learning were independently performed. Results were blinded from operators and patients. Outcomes were followed up for up to 90 days for major adverse cardiovascular and renal events (MACREs). Greater than 70% stenosis or a fractional flow reserve less than or equal to 0.8 represented hemodynamically significant coronary disease. A random forest model using demographic, comorbidities, risk factors, and lab data was trained to predict CAD severity. The Random Forest Model predictive accuracy was assessed by area under the receiver operating characteristic curve with comparison to the final diagnoses made from coronary angiography.

**Results:**

Hemodynamically significant CAD was predicted by 18-point clinical data input with a sensitivity of 81% ± 7.8%, and specificity of 61% ± 14.4% by the established model. The best machine learning model predicted a 90-day MACRE with specificity of 44.61% ± 14.39%, and sensitivity of 57.13% ± 18.70%.

**Conclusion:**

Machine learning models based on routine demographics, clinical factors, and lab data can be used to predict hemodynamically significant CAD with accuracy that approximates current noninvasive functional modalities.

**Key Findings**

• Demographics, clinical factors, and laboratory data can be used to predict hemodynamically significant coronary artery disease (CAD) using a machine learning algorithm.
• A random forest model was established with a predictive accuracy comparable to current noninvasive functional modalities in CAD diagnosis.
• Machine learning could play a role in optimizing CAD diagnosis, reducing unnecessary procedures, and improving patient safety and healthcare efficiency.

## Introduction

In the United States, coronary artery disease (CAD) is the leading cause of death, 1 in 6 yearly, for men and women, costing billions of dollars annually.^1^ The economic and public health burden that CAD has on society warrants change in the way we stratify and optimize diagnosis.^2^^,^^3^ Specifically, optimizing the diagnostic process of hemodynamically significant CAD presents great opportunities in improving population health outcomes, healthcare efficiency, and cost reduction.

Despite optionality, significant limitations exist for noninvasive functional cardiac tests in terms of cost-effectiveness and accuracy.^4^ Noninvasive functional cardiac tests used to detect hemodynamically significant CAD for clinical decision-making include nuclear myocardial perfusion study, stress electrocardiography, stress echocardiogram, and stress magnetic resonance imaging. The sensitivity and specificity for those tests in patients with elevated risk for CAD ranges from 62% to 90% and 68% to 91%, respectively,^4^ while the sensitivity and specificity in the general population ranges from 67% to 90% and 46% to 89%, respectively.^4^ For example, stress electrocardiography has a sensitivity of 62% and specificity of 46% in the suspected CAD population; stress echocardiography has an overall sensitivity of 84%–87% and specificity of 72%–77%; single photon emission computed tomography myocardial perfusion study has an overall sensitivity of 83%–85% and specificity of 77%–79%.^4^ Increasing the sensitivity and specificity of each test ought to go hand in hand with reduction in cost and increase in affordability and accessibility.

The overall sensitivity and specificity in noninvasive functional cardiac tests has room for improvement. It is not uncommon to have patients with symptoms and positive myocardial perfusion imaging suggestive of clinically significant CAD who actually have angiographically normal coronary arteries.^5^ Further, the Women’s Ischemic Syndrome Evaluation study (n >500,000, 2009) reports approximately 48% of women and 17% of men who underwent left heart catheterization had no hemodynamically significant CAD.^5^^,^^6^ This discrepancy was represented previously in Patel and colleagues,^7^ where the rate of patients without history of CAD undergoing cardiac catheterization only showed marginal improvement with use of positive noninvasive tests in finding obstructive disease (36% and 41%, respectively).

Machine learning has emerged as a highly effective tool in prediction and decision-making, and thus could prove to be an alternative or adjunctive risk-free and cost-friendly tool for CAD prediction. Machine learning algorithms improve the specificity and sensitivity for prediction of cardiovascular events and mortality, independent from or adjunctive with current available noninvasive testing.^8^^,^^9^ As a proof of concept, we present the first prospective pilot cohort study assessing the ability of machine learning in predicting hemodynamically significant CAD.

## Methods

The study protocol was approved by the institutional review board at UNC Chapel Hill. All enrolled patients provided written informed consent. Consecutive patients undergoing cardiac catheterization between March 17, 2015, and July 15, 2016, at UNC Chapel Hill were screened for comorbidities and CAD risk factors. In this single-center, prospective, observational study,185 patients at least 18 years of age were selected with at least 2 of following risk factors: congestive heart failure, chronic kidney disease (eGFR [glomerular filtration rate] 15–60 mL/min/1.73 m^2^), diabetes diagnosis, age >75 years old. The patients with the following presentations were excluded: acute coronary syndrome, any type of shock, active infection, end-stage renal disease on dialysis, active chemotherapy, and history of heart transplant.

Invasive coronary angiography was performed using standard techniques. CAD prediction by preliminary machine learning models was withheld from both operators and patients. The primary endpoint was hemodynamically significant CAD on coronary angiography, defined as greater than 70% stenosis by visual estimation or fractional flow reserve ≤0.8. Patients underwent additional postprocedure laboratory tests and 90-day follow-up for secondary endpoints for major adverse cardiovascular and renal events (MACREs). MACREs are defined as all-cause mortality, myocardial infarction, repeat revascularization, contrast-induced acute kidney injury, and renal replacement therapy. All endpoints were predicted by a machine learning model with inputs from routine demographics, clinical factors, and laboratory data.

The goal of machine learning is to predict endpoints with these variables. Based on current published risk factors of CAD, 18 variable data points were selected from routine demographics, clinical factors, and laboratory data, as shown in Table 1. In 3 of the 18 variables (most recent left ventricular ejection fraction, most recent eGFR, most recent creatinine), missing data points were present. Median imputation, a common approach for missing data handling in machine learning algorithms, was used to balance the missing data. Median imputation replaces occurrences of missing values (“NA”) within a variable with the median or mean of said variable. Each variable’s importance was computed based on χ^2^ statistics (Supplemental Table 1) and only the most statistically significant variables were used for random forest model fitting (Table 1).

**Table 1:** Baseline patient characteristics

Characteristic	Data (n = 185)
Age (years) ± SD	72 ± 11
Sex, n (%)	
Male	119 (64%)
Female	66 (36%)
CAD risk factors, n (%)	
BMI	32 ± 8
DM	126 (68%)
CKD	121 (65%)
HTN	179 (96%)
Stroke	37 (20%)
RAS	11 (5.9%)
PAD	55(30%)
sCAD	123 (66%)
HFrEF	41 (22%)
HFpEF	52 (28%)

BMI = body mass index; CAD = coronary artery disease; CKD = chronic kidney disease; DM = diabetes mellitus; HFpEF = heart failure with preserved ejection fraction; HFrEF = heart failure with reduced ejection fraction; HTN = hypertension; PAD = peripheral arterial disease; RAS = renal arterial stenosis; sCAD = suspected CAD (by calcium score/computed tomography chest without contrast).

**Figure 1:**
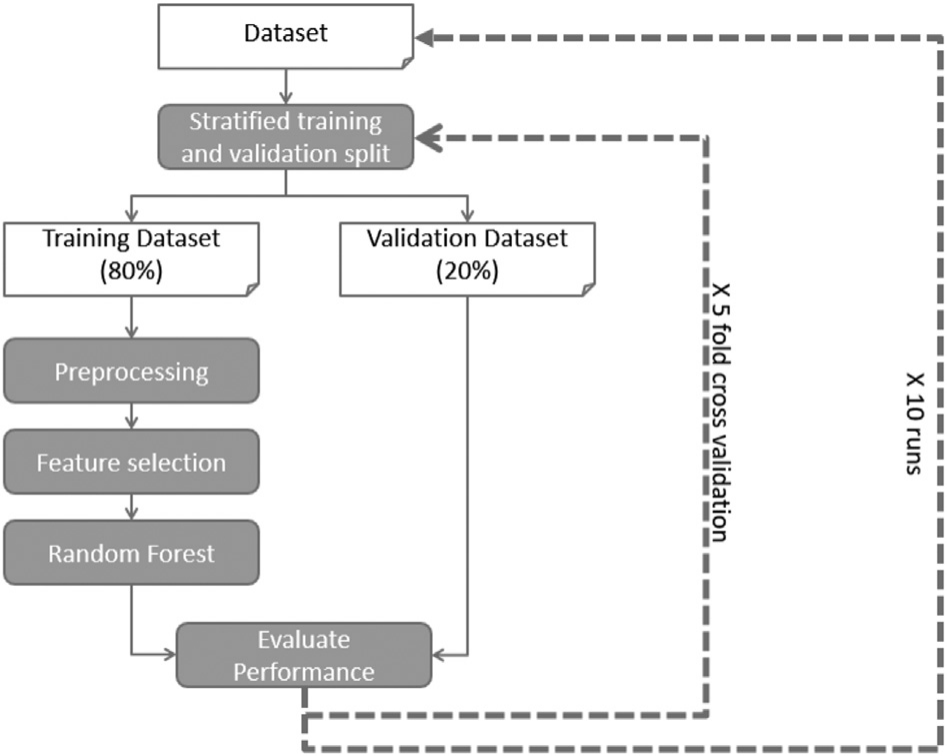
Machine learning algorithm. Using random forest model, we made 10 runs of 5-fold stratified cross-validation for performance evaluation. For each run, the dataset is randomly divided into 5 equal 5-folds, each with approximately the same number of classes. Five validation experiments are then performed, with each fold used in turn as validation set and the remaining 4 folds as the training set. This process was repeated 10 times, resulting in 50 trained random forest models, and the average validation performance across all the models was reported.

Using random forest modeling, 10 runs of 5-fold stratified cross-validation for performance evaluation were executed and were implemented in Python based on the library tool set called sklearn.^10^ For each of the 10 runs, the dataset was randomly divided into 5 equal 5-folds, until each matrix contained approximately the same number of classes. The next step was validating the reproducibility of outcomes. Five validation experiments were performed, with each fold used in turn as the validation set and the remaining 4 folds as the training set. This process was repeated 10 times, such that overall, 50 random forest models were trained. The average validation performance across all the models is reported and the entire machine learning algorithm is shown in Figure 1. Hyper-parameters like data imputing strategy, number of important features to select, and random forest classifier parameters, as shown in Supplemental Table 2, required refinement. Hyper-parameters were selected empirically.

For primary endpoint, CAD was divided into 4 classes: no significant CAD, hemodynamically significant 1-vessel disease, hemodynamically significant 2-vessel disease, and hemodynamically significant 3-plus-vessel disease. Two model-building strategies were used to predict the primary endpoint: multi-class machine learning models to predict all 4 classes simultaneously, and a single-class binary classifier machine learning model to predict “hemodynamically significant CAD” vs “no significant CAD.” In modeling the secondary endpoint MACREs, only the single-class strategy was employed. In addition to computing the sensitivity, specificity, precision, and F1 score (harmonic mean of precision and recall), area under the receiver operating characteristic curve of “vessel disease” class was also computed.

The performance of the machine learning prediction algorithms, estimated by the validation set in each cross-validation run, was assessed by sensitivity, specificity, and F1 score. For all analysis, area under curve of the receiver operating characteristic curve (ROC) is calculated and compared against the final diagnoses made from coronary angiography. Similar machine learning models were trained in an attempt to predict 90-day MACREs.

## Results

### Basic characteristics

A total of 185 patients were recruited during the pilot cohort study, with 119 (64%) male and 66 (36%) female patients. The baseline characteristics of the study population are described in Table 1. Additionally, the distribution of potential CAD risk factors, including body mass index (BMI), diabetes, chronic kidney disease, renal arterial stenosis, hypertension, heart failure with reduced ejection fraction, and heart failure with preserved ejection fraction, are listed in Table 1. There were no patient drop-outs to be accounted for in the analysis, nor any missing values in the primary endpoint and secondary endpoint. A total of 125 patients had hemodynamically significant CAD and 47 patients had MACREs. A total of 9 patients had died at 90-day follow-up.

### Primary endpoints

#### Multi-class strategy

**Figure 2:**
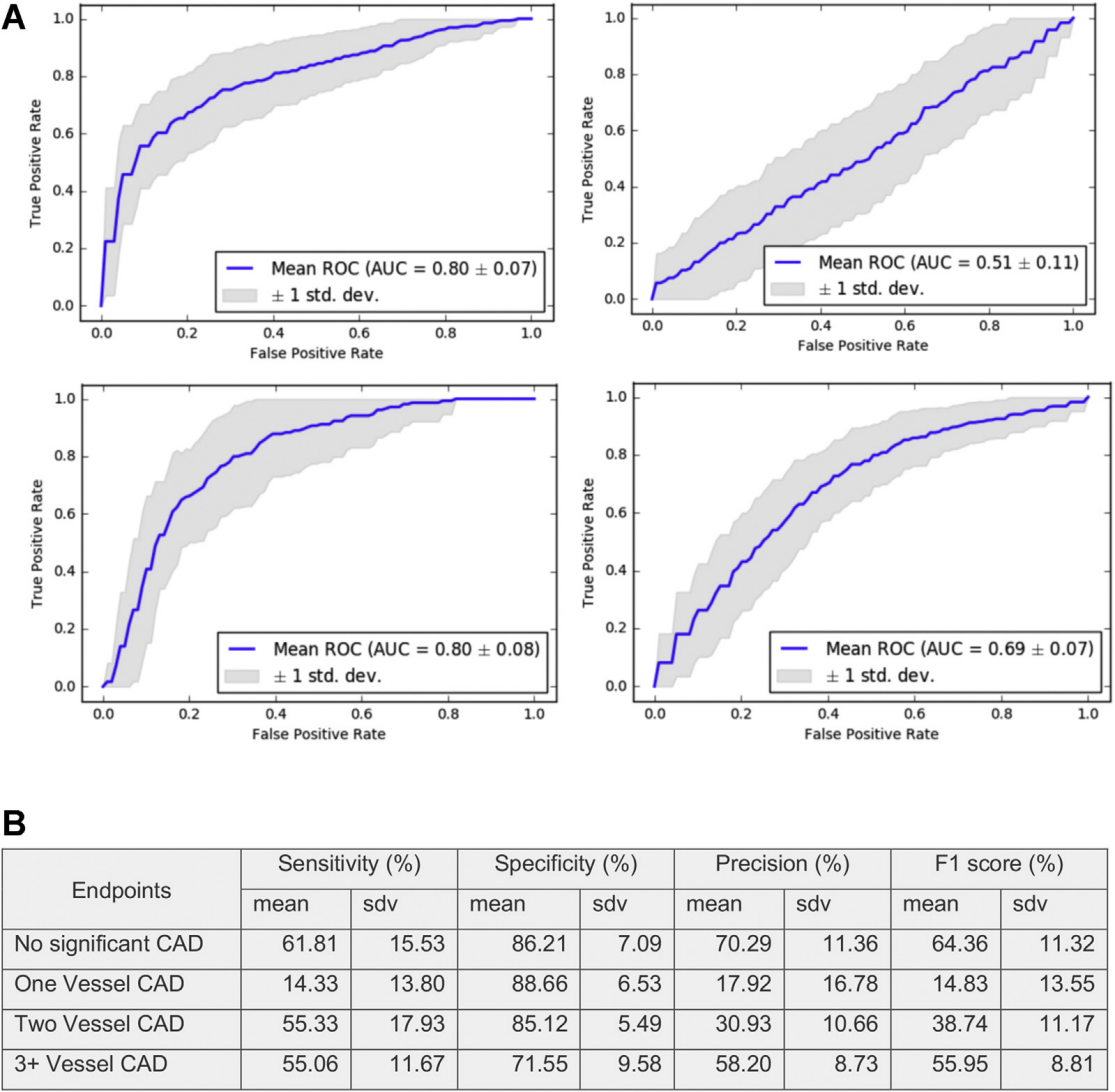
Performance of random forest model in predicting hemodynamically significant coronary artery disease (CAD) by multiclass strategy. **A:** Receiver operating characteristic curves (ROCs) for multiclass strategy in predicting “No significant CAD” (left upper panel), “hemodynamically significant 1-vessel CAD” (right upper panel), “hemodynamically significant 2-vessel CAD (left lower panel), and “hemodynamically significant 3-plus-vessel CAD (right lower panel). **B:** The performance of the machine learning model was assessed by sensitivity, specificity, precision, and F1 score. AUC = area under the curve; sdv = standard deviation.

For primary endpoints, we first used a multi-class strategy for analysis. The sensitivity, specificity, precision, and F1 scores to predict each of 4 primary endpoints (“no significant CAD,” “hemodynamically significant 1-vessel CAD,” “hemodynamically significant 2-vessel CAD,” “hemodynamically significant 3-plus-vessel CAD”) are shown in Figure 2B. Assuming 1-vs-all classification, the equivalent ROCs are shown in Figure 2A. The average and standard deviation of data point importance ranking for the learned models are listed in Table 2.

**Table 2:** Data point importance ranking from learned models in single-class and multiclass experiments

Single-class experiments			Multiclass experiments		
Data points	Importance mean	Importance Sdv	Data points	Importance mean	Importance Sdv
sCAD	0.27	0.035	Age	0.18	0.023
Age	0.14	0.026	HFrEF	0.15	0.029
BMI	0.14	0.026	Most recent eGFR	0.14	0.025
Most recent eGFR	0.13	0.021	sCAD	0.13	0.023
Most recent EF	0.07	0.016	BMI	0.12	0.036
PAD	0.07	0.020	Most recent EF	0.09	0.014
Previous cath	0.07	0.022	HFpEF	0.07	0.040
Anemia	0.03	0.020	PAD	0.04	0.011
Sex	0.03	0.022	Pulmonary edema	0.04	0.015
CKD	0.02	0.019	Previous cath	0.03	0.008
Stroke	0.02	0.015	Sex	0.004	0.013
Pulmonary edema	0.003	0.009	Anemia	0.003	0.011
RAS	0.002	0.004	CKD	0.003	0.010
DM	0	0	Stroke	0.002	0.009
HTN	0	0	HTN	0	0
HFrEF	0	0	DM	0	0
Most recent creatinine	0	0	Most recent creatinine	0	0
HFpEF	0	0	RAS	0	0

BMI = body mass index; Cath = catheterization; CKD = chronic kidney disease; DM = diabetes mellitus; EF = ejection fraction; HFpEF = heart failure with preserved ejection fraction; HFrEF = heart failure with reduced ejection fraction; HTN = hypertension; PAD = peripheral arterial disease; RAS = renal arterial stenosis; sCAD = suspected coronary artery disease; Sdv= standard deviation.

#### Single-class strategy

**Figure 3:**
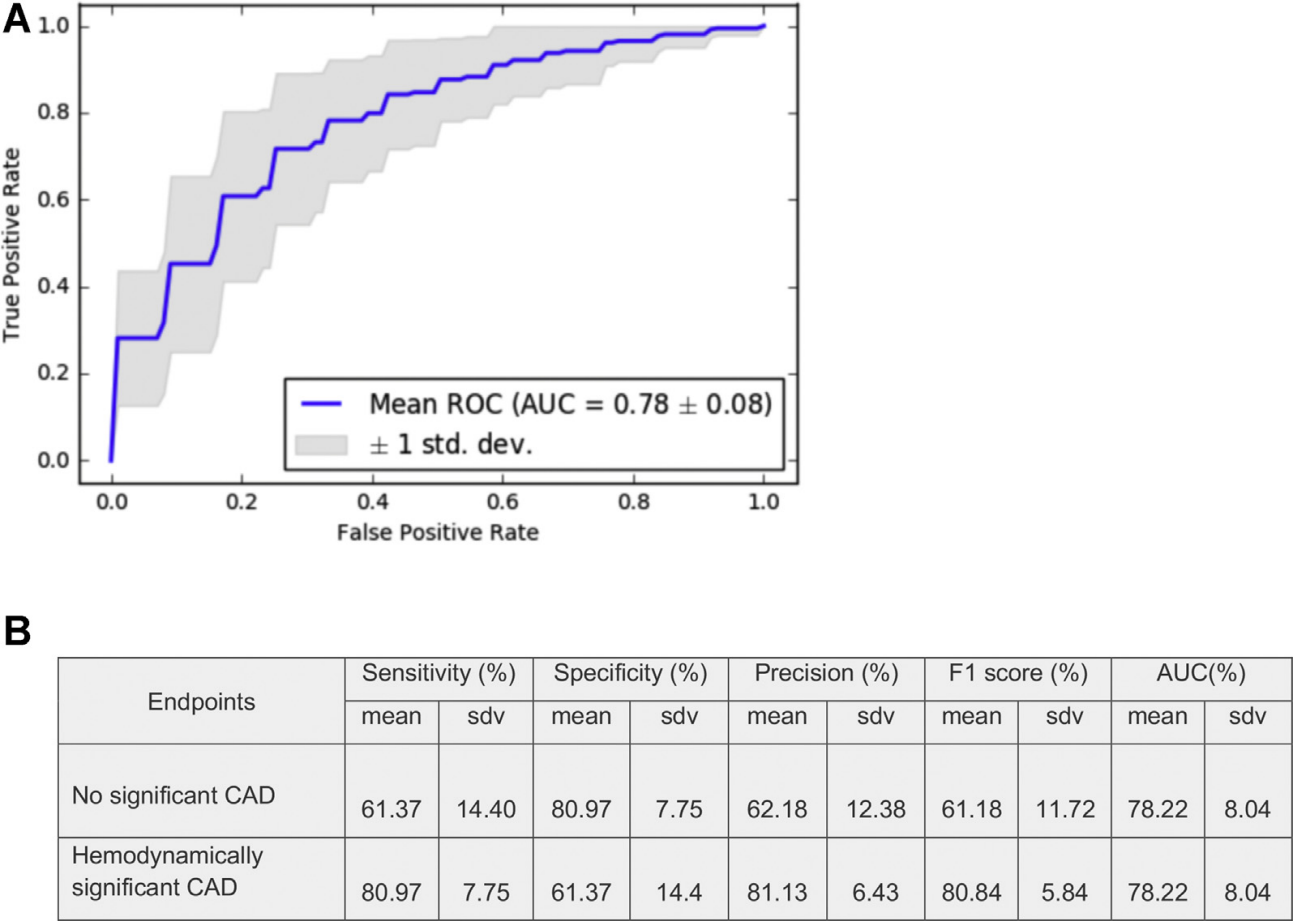
Performance of random forest model in predicting hemodynamically significant coronary artery disease (CAD) by single-class strategy. **A:** Receiver operating characteristic curves (ROCs) for single-class strategy in predicting any hemodynamically significant CAD. **B:** The performance of the machine learning model was assessed by sensitivity, specificity, precision, and F1 score. AUC = area under the curve; Sdv = standard deviation.

The random forest model was generated using single-class strategy (Figure 3B). The sensitivity and specificity for “no significant CAD” prediction was 61.37% and 80.97%, respectively. The sensitivity and specificity of “hemodynamically significant CAD” prediction was 80.97% ± 7.75% and 61.37% ± 14.4%, respectively. The precision scores were 62.18% ± 12.38% and 81.13% ± 6.43% for “no significant CAD” and “hemodynamically significant CAD,” respectively. The ROCs of the mean and standard deviation of all validation folds for both “no significant CAD” and “hemodynamically significant CAD” classes are shown in Figure 3A. The average and standard deviation of data point importance ranking for the learned models are listed in Table 2.With multiclass strategy, we achieved 51.14% micro average F1 score with 6.75% standard deviation across 10 runs of 5-fold validation. With single-class strategy, we achieved 74.47% micro average F1 score with 7.58% standard deviation. Corresponding ROC score is 78.22%. It is evident that the single-class models have overall better performance, likely owing to the larger patient number in the combined CAD dataset. Top-ranked data points (>10% importance) for both multiclass and single-class models have similar outcomes. They are age, suspected CAD, BMI, and most recent eGFR. The most significant change of data point rank between the multi-class and single-class model is heart failure with reduced ejection fraction. It is the second most significant feature in the multiclass model but has no significance in the single-class model.

### Secondary endpoints

For secondary endpoints, we used similar single-class strategy for machine learning modeling in predicting 90-day MACREs, defined as all-cause mortality, myocardial infarction, repeat revascularization, contrast-induced acute kidney injury, and renal replacement therapy. The best machine learning model was only able to predict 90-day MACREs with specificity of 44.61% ± 14.39% and sensitivity of 57.13% ± 18.70%. Among all variables examined, there were no data points correlated with MACREs in a statistically significant manner, likely owing to the low occurrence. Suspected CAD, BMI, and most recent ejection fraction appear to be top-ranked factors in the best model, but none of these had an importance mean above 0.09.

## Discussion

In this prospective, pilot cohort study, we generated a machine learning model using routine data like demographics, clinical factors, and laboratory data to predict hemodynamically significant CAD with accuracy comparable to current noninvasive functional testing modalities. With the single-class strategy, the trained random forest model predicted hemodynamically significant CAD with a sensitivity of 81% ± 7.8% and specificity of 61% ± 14.4%. This approximates the accuracy of some noninvasive functional cardiac test modalities in suspected CAD population with relatively high pretest probability^4^ (62%–90% in sensitivity and 68%–91% in specificity) at a fraction of the cost.

Two major limitations of this study are generalizability (eg, small sample size, single institution study) and scope (eg, selection for relatively high-risk population). As such, any comparisons in predictive accuracy to current noninvasive functional cardiac test modalities should be made under similar conditions (eg, high-risk population). Increasing generalizability and affirming validity of this model requires use of a larger sample size. The current machine learning model will improve with training on a larger cohort, retrospective or prospective, as substantially more data and variables have the potential to further fine-tune the accuracy in outcome prediction. The cost of using machine learning–based algorithms like random forest model on large databases is the extended run time. Nonetheless, further data training and optimization of the model may provide an alternative accurate and cost-friendly tool for CAD diagnosis and clinical decision-making.

The machine learning model, with further data training, could be a powerful tool used in conjunction with noninvasive cardiac testing not only to reduce the unnecessary invasive procedures, but also to increase the rate of detection in patients who are truly at risk of myocardial infarction. Large studies^5^^,^^6^ have shown that 30%–40% of people who undergo left heart catheterization every year have no hemodynamically significant CAD. Conversely, approximately half of myocardial infarctions and strokes occur in people who are not predicted to be at risk of cardiovascular disease.^11^ A positive noninvasive test only marginally increased the rate of identifying obstructive disease in these patients from 36% to 41%.^7^ The findings of this pilot study alone suggest that the public health and economic benefits of current or similar machine learning models could be substantial. In our study, all machine learning models explored failed to predict 90-day MACREs accurately. This could be partially due to limited sample size, population selection, and low event rate in the current study cohort. Some clinical factors such as suspected CAD, BMI, and most recent ejection fraction appear to be top-ranked variables in the best model but none of these had any meaningful importance, therefore future large-scale studies are needed in order to determine if machine learning models could be useful tools in clinical outcome prediction.

## Conclusion

We examined the ability of machine learning to predict CAD severity in this single-center, prospective, pilot cohort study. A random forest machine learning model was established based on routine demographics, clinical factors, and laboratory data to predict hemodynamically significant CAD with accuracy comparable to current noninvasive functional testing modalities. There are significant limitations to the study, and the population in which this model can be applied should be selective at the current stage. Further data training and optimization of the model with a larger clinical study may provide a more accurate and cost-friendly complementary tool for CAD diagnosis and clinical decision-making, which in turn may play an important role in reducing unnecessary invasive angiography, improving patient safety and outcome as well as optimizing healthcare efficiency in this patient population.
