# Supplementary material for: A machine learning model in predicting hemodynamically significant coronary artery disease: A prospective cohort study
Source: Cardiovasc Digit Health J. 2022 Mar 7;3(3):112–7. doi: 10.1016/j.cvdhj.2022.02.002 (PMC9204796; doi:10.1016/j.cvdhj.2022.02.002)
Supplement: Supplemental Tables 1 and 2 [file mmc1.pdf]

Supplemental Table 1. Data point description

feature	unit	chi-square test p-value
Age		0.0006
sCAD*	yes/no	0.0012
Most recent eGFR		0.0013
PAD	yes/no	0.0134
BMI		0.0195
Previous Cath	yes/no	0.0205
Gender	male/female	0.0426
Most recent EF		0.0966
CKD	yes/no	0.1226
Stroke	yes/no	0.1972
Anemia	yes/no	0.2010
Pulmonary Edema	yes/no	0.4487
RAS	yes/no	0.4962
HFpEF	yes/no	0.6032
HFrEF	yes/no	0.6589
Most recent creatinine		0.7457
DM	yes/no	0.7931
HTN	yes/no	0.8079

sCAD*: suspected CAD by calcium score/CT chest without contrast

Supplemental Table 2. Hyper parameters for random forest training process

Parameter name	Value
Imputing strategy	Most Frequent
Number of important features	10
Max tree depth	3
Number of trees	100
Max features	auto
Class weight	Balanced
